# Generalized Frequency Division Multiplexing-Based Low-Power Underwater Acoustic Image Transceiver

**DOI:** 10.3390/s22010313

**Published:** 2021-12-31

**Authors:** Chin-Feng Lin, Cheng-Fong Wu, Ching-Lung Hsieh, Shun-Hsyung Chang, Ivan A. Parinov, Sergey Shevtsov

**Affiliations:** 1 Department of Electrical Engineering, National Taiwan Ocean University, Keelung 20224, Taiwan; smartntou@gmail.com (C.-F.W.); healthcarentou@gmail.com (C.-L.H.); 2 Department of Microelectronics Engineering, National Kaohsuing University of Science and Technology, Kaohsuing 81157, Taiwan; 3 I. I. Vorovich Mathematics, Mechanics, and Computer Science Institute, Southern Federal University, 344090 Rostov-on-Don, Russia; parinov_ia@mail.ru; 4 Head of Aircraft Systems and Technologies Lab at the South Center of Russian Academy of Science, 344006 Rostov-on-Don, Russia; sergnshevtsov@gmail.com

**Keywords:** GFDM, low-power, image transceiver, BER, simulation of transmission

## Abstract

In this paper, a low-power underwater acoustic (UWA) image transceiver based on generalized frequency division multiplexing (GFDM) modulation for underwater communication is proposed. The proposed transceiver integrates a low-density parity-check code error protection scheme, adaptive 4-quadrature amplitude modulation (QAM) and 16-QAM strategies, GFDM modulation, and a power assignment mechanism in an UWA image communication environment. The transmission bit error rates (BERs), the peak signal-to-noise ratios (PSNRs) of the received underwater images, and the power-saving ratio (PSR) of the proposed transceiver obtained using 4-QAM and 16-QAM, with perfect channel estimation, and channel estimation errors (CEEs) of 5%, 10%, and 20% were simulated. The PSNR of the received underwater image is 44.46 dB when using 4-QAM with a CEE of 10%. In contrast, PSNR is 48.79 dB when using 16-QAM with a CEE of 10%. When BER is 10^−4^, the received UW images have high PSNR values and high resolutions, indicating that the proposed transceiver is suitable for underwater image sensor signal transmission.

## 1. Introduction

Underwater acoustic (UWA) communication plays a significant role in ocean exploration and UWA sensor network monitoring. The Internet of Underwater Things (IoUWT) and sixth-generation (6G) communication transmission schemes have significantly contributed to increasing the underwater data throughput and transmission power efficiency of UWA communication [1,2]. However, designing a high-speed, reliable, and low-power UWA communication system is challenging. Zhou et al. [3] derived underwater statistical propagation characteristics using closed-form probability density functions for calculating the angle of departure and arrival. The spatial and frequency correlation functions of two different UWA propagation paths were explored. The UWA signal attenuation was found to increase with increasing signal frequency. The underwater channel offers time-varying multipath propagation, and the acoustic wave has a low transmission speed. The simulations are an important step for the development of UWA communication systems. Morozs et al. [4] demonstrated the importance of simulations in the development and performance evaluation of UWA communication systems and provided a clear insight into the simulation approach to efficient UWA communication system implementation. The UWA channel model plays a significant role in the UWA communication system simulation. The simulations provide a reference design, as the system can be designed under different assumptions for a more comprehensive result, e.g., via the design and adjustment of different parameters (e.g., transmission power).

Zanaj et al. [5] simulated the communication performance of the UWA channel capacity parameter in an UWA sensor network. However, data communication in underwater environments is difficult due to time-varying channel parameters. An accurate simulation approach is required to replicate the behavior of UWA sensor networks in a real scenario, and improve the transmission performance of UWA sensor networks. Kari et al. [6] proposed an adaptive robust channel estimation mechanism for highly time-varying UWA communication channels with multipath propagation, large delay spreads, and frequency-dependent transmission loss. Adaptive filtering techniques, with a minimization logarithmic cost function, increased the convergence rate of the channel estimators. Orthogonal frequency division multiplexing (OFDM)-based UWA communication transmission solutions can combat the effects of long and time-varying channel impulse responses in an underwater environment. The bandwidth efficiency, reliability, latency, transmission throughput, and changing underwater propagation channel are critical parameters for UWA communication system implementation. Emerging UWA sensor network applications rely on physical layer technology due to its high throughput, reliability, and energy efficiency. Song et al. [7] aimed to explore new directions for next-generation underwater acoustic modems. Simulation methods are necessary to explore the design aspects of underwater acoustic communication, and the sea experimentation cost can be reduced. Liu et al. [8] focused on the physical fundamentals and engineering implementations for efficient data transmission via mobile communication using physical waves in UWA sensor networks. Acoustic waves are the most widely used waves, due to the low signal attenuation of sound in water. The challenges of the UWA communication system include long latency, low bandwidth and high time-varying channel characteristics. MATS 3G has been used in underwater communications for decades, with impressive antenna sizes at the transmitters and loop antennas at the receivers [9].

Ahmad et al. [10] adopted the downlink power allocation strategy using an OFDM technique for UWA communication networks. The block error rates with different signal-to-noise ratio (SNR) values were presented, and throughput performances for with and without power assignments (PAs) were compared. Cheon et al. [11] proposed a power allocation technology for non-orthogonal multiple access (NOMA) in UWA sensor networks. The feasibility of the NOMA scheme in an underwater channel with distance-/frequency-dependent attenuation and frequency-dependent ambient noise was explained. In addition, the performance degradation of the sum-rate maximizing power allocation strategy for downlink underwater NOMA was investigated. NOMA is a possible multiple-access scheme for 6G mobile communication, and GFDM is a highly flexible NOMA [12]. Luna et al. [13] evaluated the BER performance of OFDM, generalized frequency division multiplexing (GFDM), and frequency-shift keying (FSK) UWA modems over a UWA channel model. Simulation results show that UWA communication systems using GFDM have a better BER performance than those using OFDM for the same underwater transmission environments. The advanced wireless communication technology of the future aims to achieve high flexibility, high bandwidth, low latency, and high spectral and energy efficiency. GFDM, using filters with overlapping subcarriers to reduce the spectral broadening of the original signal, is a more flexible communication scheme to achieve the above aims. Deepthi et al. [14] discussed a generalized digital multiple-carrier transceiver design concept, and adopted the channel bank multi-branch multicarrier design in the GFDM physical layer.

Michailow et al. [15] demonstrated the BER and symbol error (SE) performance over different channel models. The modulation of independent time–frequency domain blocks was adopted in the GFDM scheme to achieve low latency. GFDM is an advanced physical layer, having high reliability, high energy efficiency, and robust throughput. GFDM provides highly flexible time–frequency block structures using inverse fast Fourier transform (IFFT) and multi-IFFT-symbol (subsymbols) methods for dynamic spectrum allocation. Each block structure had *MK* samples with *K* subcarriers carrying *M* subsymbols. An entire block with multiple subsymbols adds a single cyclic prefix (CP), longer than the multipath channel impulse response, to combat multipath channel fading and achieve better spectral efficiency in the GFDM system. The subcarriers were filtered using a root-raised cosine (RRC) filter with circular shifts in the time and frequency domains to reduce out-of-band (OOB) emissions. The next-generation advanced communication techniques face different challenges when used in new applications. Nimr et al. [16] demonstrated that the OFDM scheme has difficulty fulfilling demanding requirements. Synchronization, channel estimation algorithms and MIMO techniques for GFDM were demonstrated in detail, and were found to be a suitable transmission method for advanced communication technologies of the future. However, compared to the OFDM approach, GFDM implementation requires more complex multiplications per data symbol to account for the overlapping subsymbols in the time–frequency domain.

Tadayon et al. [17] proposed a low-complexity and high-transmission data rate underwater acoustic OFDM technology. The acoustic OFDM system used differentially coherent detection with a small pilot overhead and experimental data transmitted over a 3–7 km shallow-water channel in the 10.5–15.5 kHz acoustic band. Radosevic et al. [18] demonstrated design considerations and experimental results for an adaptive OFDM underwater modem. The adaptive binary phase-shift keying (BPSK), quadrature phase-shift keying (QPSK), 8 phase-shift keying (8PSK), 16-quadrature amplitude modulation (16-QAM), and power adjustment approach were integrated into the adaptive OFDM underwater communication scheme. Kochanska et al. [19] conducted UWA communication tests using an OFDM-based data transmission method in a shallow water environment at Wdzydze Lake. The OFDM signal within a band of 5 kHz was transmitted in the frequency range between 27.5 to 32.5 kHz. The underwater OFDM-based system had a carrier frequency of 30 kHz, and used the binary phase-shift keying (BPSK) modulation strategy. Lin et al. [20] proposed a UWA multimedia communication scheme with 2400 transmission modes to combat the time-varying multipath UWA channel environment. The orthogonal variable spreading factor scheme, gold sequence scramble code, direct mapping (DM), or space–time block code multi-input multi-output (MIMO) transmission strategy, OFDM, BPSK, or quadrature phase-shift keying adaptive modulation, a convolution channel code with rates of 1/2 and 1/3, and a power assignment mechanism, were integrated into the proposed UW OFDM-based system. Amini et al. [21] developed the filterbank multicarrier (FBMC) technique in doubly dispersive UWA communication channels, and a filterbank prototype filter with a novel cost optimization function to achieve robust signal-to-interference-plus-noise ratios and BER performances. Lin et al. [22,23] explored an FBMC-based UWA multimedia communication scheme incorporating single-input single-output (SISO) and DM MIMO transmission mechanisms, (2000, 1000) low-density parity-check (LDPC) encoders, adaptive BPSK or offset quadrature amplitude modulations, and a power assignment method. Sun et al. [24] proposed a filtered multitone (FMT) modulation-based UWA transmission system with low-complexity channel-estimation (CE)-based minimum mean square error (MMSE) turbo equalization, and evaluated the symbol error rate (SER) performances. Simulation results show better transmission performance with higher throughput than the FMT modulation UWA transmission with traditional MMSE adaptive equalization.

Murad et al. [25] evaluated the performance of a GFDM transceiver in a high-data-rate UWA transmission channel in terms of the spectral efficiency, peak-to-average power ratio, SER, and complexity of the proposed GFDM transceiver. Wu et al. [26] analyzed the peak-to-average power ratio (PAPR) performance of a GFDM system with a pulse-shaping filter, and compared it with that of the OFDM. Hebbar et al. [27] developed a non-orthogonal multicarrier scheme, GFDM, for reliable and efficient underwater transmission systems, and evaluated its BER performance in additive white Gaussian noise (AWGN) and Rayleigh fading UW channels. The advantages of a GFDM physical layer design, such as low transmission BER performance, low computational complexity, and less bandwidth overhead, make GFDM a suitable transmission scheme in underwater communication.

The remainder of this paper is organized as follows: In Section 2, the proposed GFDM-based UWA image transmission technology is briefly described. In Section 3, the simulation results of the proposed transceiver are presented and the underwater image transmission scheme performance is evaluated in detail. Finally, the conclusions are presented in Section 4.

## 2. GFDM-Based Low-Power Underwater Image Transceiver Architecture

**Figure 1:**
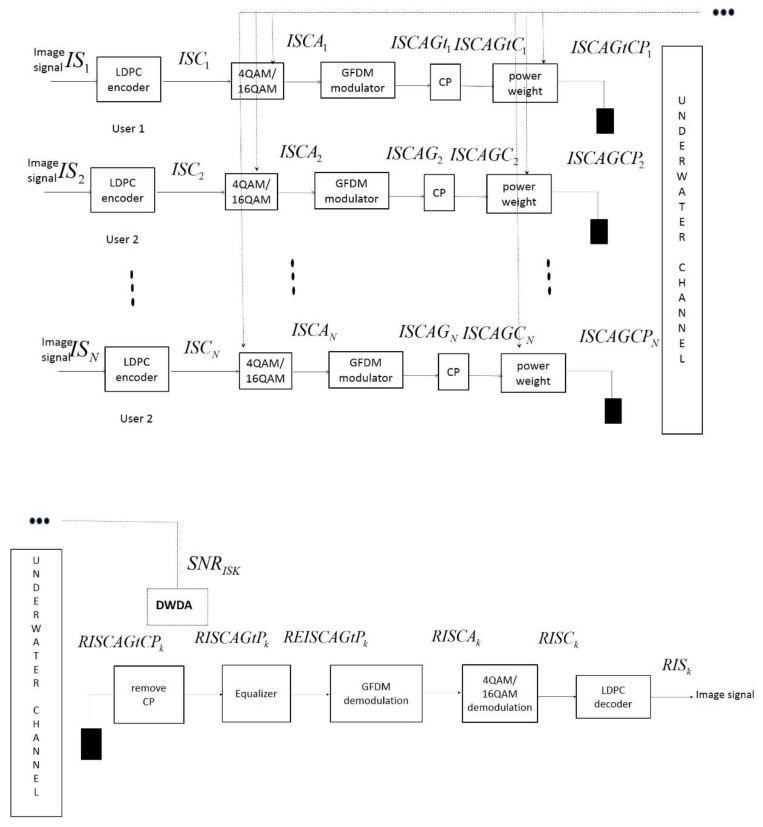
Proposed GFDM-based low power UWA image transceiver architecture.

Figure 1 shows the proposed GFDM-based low-power UWA image transceiver architecture. The technology integrates the LDPC encoders, the adaptive 4-quadrature amplitude modulation (QAM) and 16-QAM strategies, a GFDM modulator, CP modules, a power assignment mechanism (PAM), a carrier-sense multiple-access collision-avoidance (CSMA/CA) transmission method, and a double-window detection algorithm (DWDA).

The use of (2000, 1000) low-density parity-check (LDPC) codes is an advanced error-correction method employed to achieve reliable underwater communication. A (2000, 1000) LDPC code is a type of linear block code and is defined as a parity-check matrix H, containing mostly zeros (0 s) and a small number of ones (1 s). It is necessary to set (2000, 1000) × 2000 as the size of the H matrix to obtain an (2000, 1000) LDPC code, where (2000, 1000) is the number of rows, 2000 is the number of columns and 1000 is the number of message bits. The coding rate is defined by 1000/2000 (1/2) for an (2000, 1000) LDPC code. When the H matrix is considered, the number of 1 bits in a row is called the row weight and, similarly, the number of 1 bits in a column is called the column weight. In addition, the (2000, 1000) low-density parity-check (LDPC) codes with a code rate of 1/2, a row weight of 6, and a column weight of 3 were integrated into the proposed underwater acoustic image transceiver [28,29].

The CSMA/CA network access method was adopted to achieve multiuser communication. Digital image signal bit streams of the *k*th user ($IS_{k}$) are input into the (2000, 1000) LDPC encoder. The LDPC-encoded image signal bit streams of the *k*th user ($ISC_{k}$) are then input into the adaptive 4-QAM/16-QAM modules. The resulting adaptive LDPC-modulated image signal bit streams of the *k*th user ($ISCA_{k}$) are then fed into the GFDM modulator to extract the transmitting data of the *k*th user ($ISCAGt_{k}$). After adding CP, the transmission signal can be described as $ISCAGtC_{k}$, which is input into the PAM, and extracted as the transmission signal with PAM ($ISCAGtCP_{k}$).

The detailed GFDM modulation is expressed as follows:

The dimension of $ISCA_{k}$ is A×1, and comprises *B* subcarriers with *C* subsymbols, which satisfies the equation A=B×C. The vector $ISCA_{k}$ can be expressed as follows:

(1) $$ISCA_{k}={\left(ISCA_{k0}^{T},\cdots ,ISCA_{kC-1}^{T}\right)}^{T}$$

(2) $$ISCA_{k0}={\left(ISCA_{0,k0}^{T},\cdots ,ISCA_{B-1,k0}^{T}\right)}^{T}$$

(3) $$ISCA_{kc}={\left(ISCA_{0,kc}^{T},\cdots ,ISCA_{B-1,kc}^{T}\right)}^{T}$$

where ${ISCA}_{b,kc}$ is the adaptive LDPC-modulated image signal bit stream, transmitted on the *b*th subcarrier and the *c*th subsymbol by the *k*th user. $g_{b,c}[e]$ is the time and frequency transformations of the prototype filter *g*[*e*], where e denotes the sampling index.

(4) $$g_{b,c}[e]=g[(\begin{array}{ccc} \left(e-cB\right) & \mathrm{mod} & A]e^{-j2\pi \frac{b}{B}e} \end{array}$$

The transmitting data of the *k*th user $ISCAGt_{k}={\left(ISCAG_{k}[e]\right)}^{T}$

(5) $$ISCAG_{k}[e]=\sum_{b=0}^{B-1}\sum_{c=0}^{C-1}g_{b,c}[e]ISCA_{b,kc},\;e=0,1,\cdots ,A-1$$

Let

(6) $$gt_{b,c}={\left(g_{b,c}[e]\right)}^{T}ISCAGt_{k}=F\cdot ISCA_{k}$$

where the dimension of *F* is BC×BC and can be expressed as

(7) $$F=(gt_{0,0},\cdots ,gt_{B-1,0},gt_{0,1},\cdots ,gt_{B-1,C-1})$$

where $gt_{k,m}$ is the time and frequency shifted versions of $gt_{0,0}$. The relation between $ISCAGtC_{k}$ and $ISCAGtCP_{k}$ is expressed as

(8) $$ISCAGtCP_{k}=\mu_{k}ISCAGtC_{k}$$

where $u_{k}$ is the transmission power weighting factor, $u_{k}\in \{0.1,0.2,\cdots ,0.9,1\}$.

The transmission through the UW channel then can be modeled by

(9) $$RISCAGtCP_{k}=H\cdot ISCAGtCP_{k}+w_{k}$$

where $RISCAGtCP_{k}$ and $ISCAGtCP_{k}$ are the receiving and transmission image signal of the *k*th user, respectively. H denotes an N×N UW channel matrix. $w_{k}$ is the additive white Gaussian noise (AWGN) of the kth user. $RISCAGtP_{k}$ is the $RISCAGtCP_{k}$ after removing CP. After channel estimation and equalization, the output signal is represented as:

(10) $$REISCAGtP_{k}=H^{-1}RISCAGtCP_{k}$$

(11) $$REISCAGtP_{k}=\mu_{k}F\cdot ISCA_{k}+H^{-1}w_{k}$$

The GFDM demodulation output of the *k*th user can be represented as:

(12) $$RISCA_{k}=OREISCAGtP_{k}$$

(13) $$RISCA_{k}=O(\mu_{k}F\cdot ISCA_{k}+H^{-1}w_{k})$$

(14) $$RISCA_{k}=F^{-1}(\mu_{k}F\cdot ISCA_{k}+H^{-1}w_{k})$$

(15) $$RISCA_{k}=\mu_{k}ISCA_{k}+F^{-1}H^{-1}w_{k}$$

The zero forcing equation method is integrated into the proposed GFDM-based UWA image receiver. The dimension of the matrix *O* is A×A, and can be expressed as:

(16) $$O=F^{-1}$$

where $RISCA_{k}$ is input into the adaptive 4-QAM/16-QAM demodulation system, and the adaptive LDPC demodulated image signal bit streams of the *k*th user ($RISC_{k}$) are extracted as outputs. $RISC_{k}$ is input into the LDPC decoder, and the image signal bit streams of the kth user $RIS_{k}$ are received.

The double-window detection algorithm [30] is integrated into the proposed GFDM-based UWA image receiver architecture to detect the SNRs of UW sensor GFDM-based image packets. The energies of windows *q* − 1 and q are $c_{k,q-1}$ and $c_{k,q}$, respectively, for the *k*-th user, and are expressed as follows:

(17) $$c_{k,q-1}={\sum_{j=1}^{A}RISCAGtP_{k,,q-1,j}RISCAGtP_{k,q-1,j}^{*}=\sum_{j=1}^{A}\left|RISCAGtP_{k,,q-1,j}\right|}^{2}$$

(18) $$c_{k,q}={\sum_{j=1}^{A}RISCAGtP_{k,,q,j}RISCAGtP_{k,q,j}^{*}=\sum_{j=1}^{A}\left|RISCAGtP_{k,,q,j}\right|}^{2}$$

The decision parameter $d_{k}$ of the *k*th user is given as follows:

(19) $$d_{k}=\frac{c_{k,q-1}}{c_{k,q}}=\frac{R_{k}+W_{k}}{W_{k}}=SNR_{k}+1$$

where $R_{k}$ and $W_{k}$ are the sums of the signal and noise energies, respectively, across the two windows for the *k*th user. $SNR_{k}$ is the SNR of the *k*th user. The transceiver then makes a decision based on prespecified thresholds. The proposed PAM is summarized as follows:

Step 1 Select the appropriate modulation strategy to satisfy the BER requirement for image signal transmission over a UWSN;
Step 2 Assign the initial value of *μ_k_* to 5/10 for the UW sensor image packets;
Step 3 Measure the received SNR of the UW sensor image packets;
Step 4 If the measured SNR of the received UW sensor image packets exceed the threshold SNR at which the required BER for UW sensor image packets, $u_{k}$, then $\mu_{k}=u_{k}-\Delta$. Parameter Δ depends on the variation in the UW channel’s fading characteristics. If $\mu_{k}\geq 1/10$, return to Step 3; otherwise, proceed to Step 6;
Step 5 If the measured SNR of the received UW sensor image packets is less than the threshold SNR at which the required BER for UW sensor image packets is achieved, $u_{k}$ then $\mu_{k}=u_{k}+\Delta$. If $\mu_{k}\leq 1$, return to Step 3; otherwise, proceed to Step 6;
Step 6 Change the modulation strategy; return to Step 2.

When Δ is 1/10, the power assignment convergence speed is faster than when Δ is 1/30. When Δ is 1/30, the power-saving efficiency is better than when Δ is 1/10. The initial transmission power being equal to 5/10 and Δ being equal to 1/10 are better design parameters for the tradeoff consideration of power assignment convergence speed and power-saving efficiency.

## 3. Simulation Results

A MATLAB-based UWA channel model was developed by Chitre et al. [31], and the MATLAB-based UWA channel model was integrated into the simulation. The UWA channel model, having a carrier central frequency of 40 kHz, a underwater channel bandwidth of 20 kHz, and a transmission distance of 100 m, was used for simulations. The transmission distance was 100 m, the water was 14.5 m deep, and the transmitter and receiver were set 3 m and 2 m beneath the sea surface, respectively. The simulation parameters of the proposed GFDM-based low-power UWA image transceiver are listed in Table 1.

**Figure 2:**
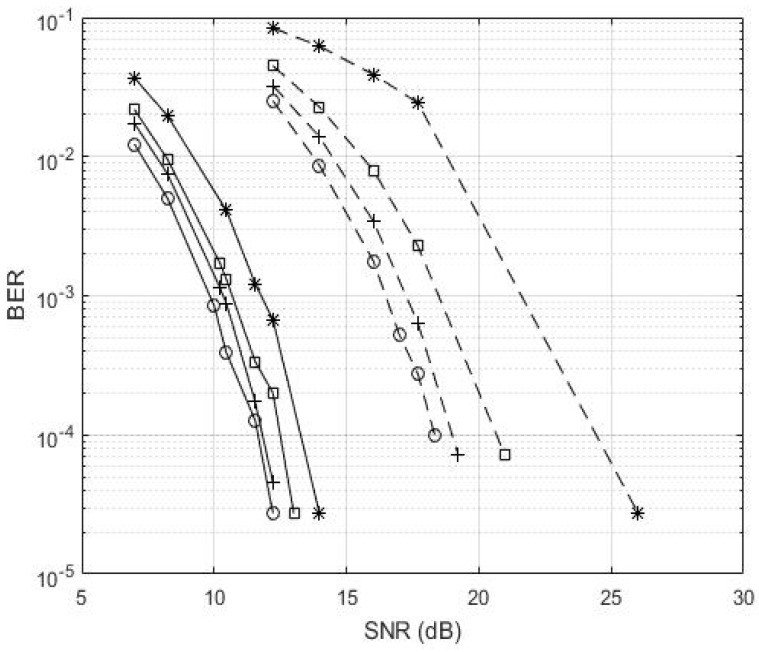
BER performance of the GFDM-based UWA image transceiver. (line: 4-QAM, dotted line: 16-QAM, o: perfect, +: CEE of 5%, □: CEE of 10%, and *: CEE of 20%).

Figure 2 shows the BER performance of the GFDM-based UWA image transceiver. When using the 4-QAM strategy, the BER values of the proposed transceivers with CEEs of 0%, and 5% with 12.22 dB SNR are $2.73\times 10^{-5}$, and $4.55\times 10^{-5}$, respectively, while the SNR values with CEEs of 10%, and 20% for BER of $2.73\times 10^{-5}$, are 13.01 dB, and 13.98 dB, respectively. In addition, the SNR increases as the CEE changes from 0% to 10%, and 10% to 20% are 0.79 dB and 0.97 dB, respectively. The proposed GFDM-based UWA image transceiver with the PCE can have the same transmission BER performance with a lower SNR than the transceiver with a CEE of 20%. When using the 16-QAM strategy, BER values with CEE of 0%, and SNR of 18.33 dB, is $10^{-4}$, while the SNR values with CEEs of 5%, and 10%, for a BER of $7.27\times 10^{-5}$, are 19.21 dB, and 20.97 dB, respectively. The SNR performance with 20% CEEs for BER of $2.73\times 10^{-5}$ is 26.02 dB. The SNR of the proposed transceiver increases as the CEE changes from 0% to 5%, and 5% to 10% are approximately 0.88 dB and 1.76 dB, respectively. The results show that the 4-QAM has better transmission BER performances than the 16-QAM in the underwater environment, while the 16-QAM has a higher transmission throughput than 4-QAM.

The image MSE (IMSE) of a g×h image is defined as follows:

(20) $$IMSE=\frac{1}{gh}\sum_{i=0}^{g-1}\sum_{j=0}^{h-1}{\left[P(i,j)-Q(i,j)\right]}^{2}$$

where *P*(*i*, *j*) and *Q*(*i*, *j*) are matrices with the pixel values of the original and received UW image signals, respectively. The peak signal-to-noise ratio (PSNR) (dB) is expressed as

(21) $$PSNR=10\log_{10}(\frac{Max{\left(I(i,j)\right)}^{2}}{IMSE})$$

**Figure 3:**
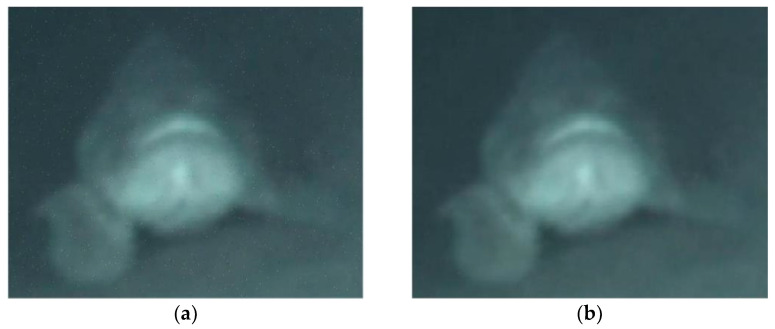
Received underwater image 1 signals using 4-QAM with the CEE of 10% and transmission BERs of 10^−3^ and 10^−4^, respectively. ((**a**) PSNR = 50.31 dB; (**b**) PSNR = 62.39 dB).

**Figure 4:**
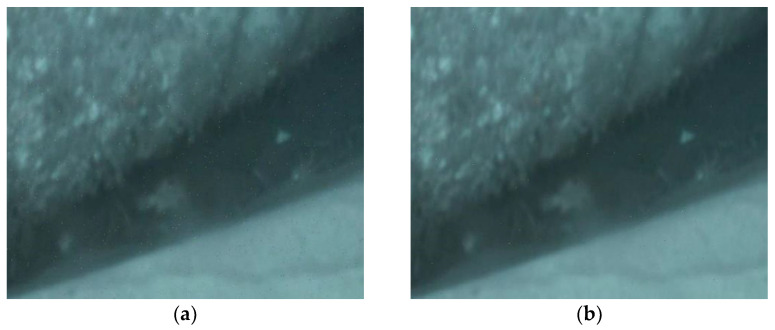
Received underwater image 2 signals using 4-QAM with the CEE of 10% and transmission BERs of 10^−3^ and 10^−4^, respectively. ((**a**) PSNR = 34.74 dB; (**b**) PSNR = 44.46 dB).

**Figure 5:**
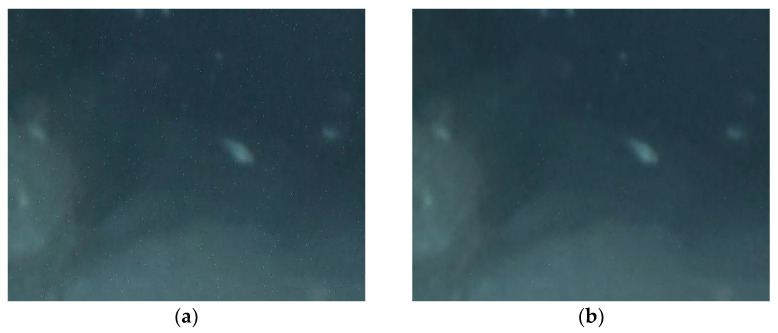
Received underwater image 3 signals using 4-QAM with the CEE of 10% and transmission BERs of 10^−3^ and 10^−4^, respectively. ((**a**): PSNR = 34.73 dB; (**b**) PSNR = 45.64 dB).

**Figure 6:**
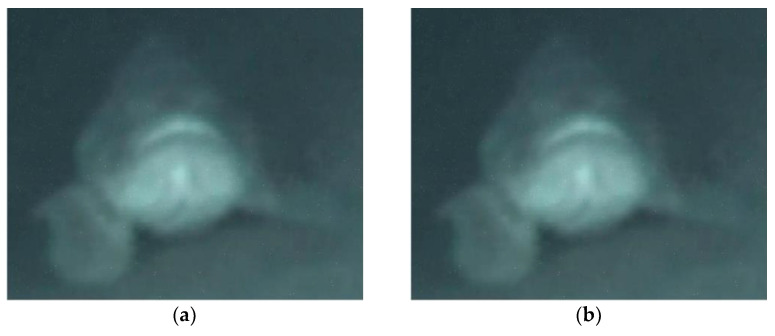
Received underwater image 1 signals using 16-QAM with the CEE of 10% and transmission BERs of 10^−3^ and 10^−4^, respectively. ((**a**) SNR = 10.46 dB, PSNR = 60.45 dB; (**b**) SNR = 12.44 dB, PSNR = 71.63 dB).

Figure 3a,b shows the received underwater image 1 signals using 4-QAM, with the transmission BERs of $10^{-3}$ and $10^{-4}$, respectively, in the GFDM-based underwater image transceiver with a CEE of 10%. The PSNRs are 50.31 dB and 62.39 dB, respectively. Figure 4a,b shows the received underwater image 2 signals using 4-QAM, with the transmission BERs of $10^{-3}$ and $10^{-4}$, respectively, in the GFDM-based underwater image transceiver with a CEE of 10%. The PSNR is 34.74 dB and 44.46 dB, respectively. Figure 5a,b shows the received underwater image 3 signals using 4-QAM, with the transmission BERs of $10^{-3}$ and $10^{-4}$, respectively, in the GFDM-based underwater image transceiver with a CEE of 10%. The PSNR is 34.73 dB and 45.64 dB, respectively. Figure 6a,b shows the received underwater image 1 signals using 16-QAM, with the transmission BERs of $10^{-3}$ and $10^{-4}$, respectively, in the GFDM-based underwater image transceiver with a CEE of 10%. The PSNR is 60.45 dB and 71.63 dB, respectively.

**Figure 7:**
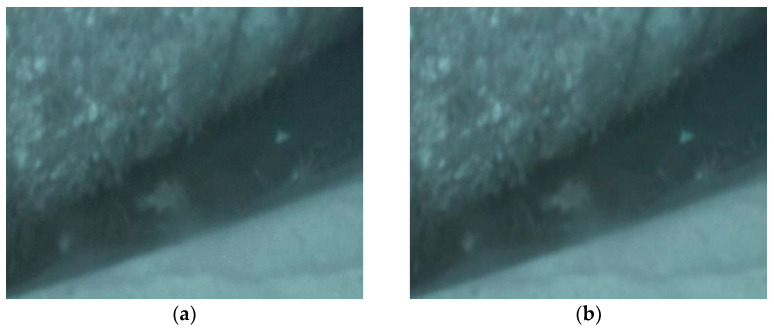
Received underwater image 2 signals using 16-QAM with the CEE of 10% and transmission BERs of 10^−3^ and 10^−4^, respectively. ((**a**) PSNR = 38.18 dB; (**b**) PSNR = 48.79 dB).

**Figure 8:**
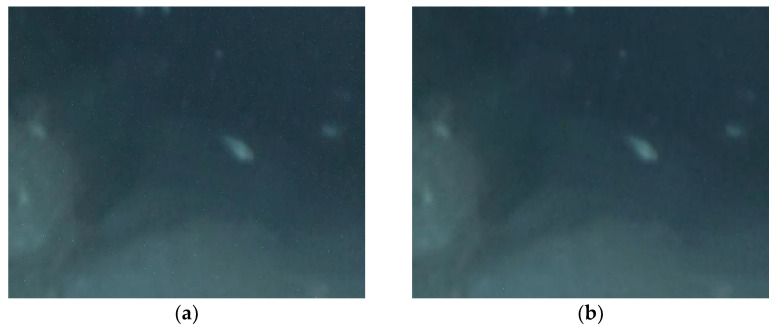
Received underwater image 3 signals using 16-QAM with the CEE of 10% and transmission BERs of 10^−3^ and 10^−4^, respectively. ((**a**) PSNR = 38.21 dB; (**b**) PSNR = 49.87 dB).

Figure 6b and Figure 7a show the received underwater image 2 signals using 16-QAM, with the transmission BERs of $10^{-3}$ and $10^{-4}$, respectively, in the GFDM-based underwater image transceiver with a CEE of 10%. The PSNR is 38.18 dB and 48.79 dB, respectively. Figure 8a,b shows the received underwater image 3 signals using 16-QAM, with the transmission BERs of $10^{-3}$ and $10^{-4}$, respectively, in the GFDM-based underwater image transceiver with a CEE of 10%. The PSNR is 38.21 dB and 49.87 dB, respectively. The simulation results show that the received underwater image 1, 2, and 3 signals with a transmission BER of $10^{-4}$, respectively, are high PSNR values, and high image resolutions. The transmission BER requirements for the underwater image signals are $10^{-4}$, indicating that low-power transmission can be achieved for underwater image communication.

The power-saving ratio (PSR) of the GFDM-based low-power UWA image transceiver for the *k*-th user is expressed as

(22) $$\begin{array}{cc} PSR_{k}=(1-\mu_{k})\times 100 & \% \end{array}$$

**Figure 9:**
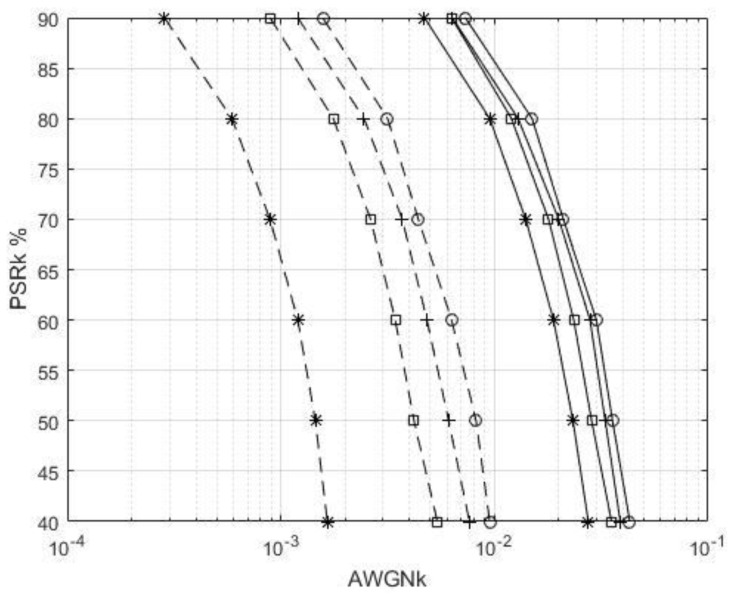
PSR performance of the GFDM-based UWA image transceiver for the *k*-th user with a BER of 10^−4^. (line: 4-QAM, dotted line: 16-QAM, o: perfect, +: CEE of 5%, □: CEE of 10%, and *: CEE of 20%).

Figure 9 shows the PSR performance of the proposed transceiver for the *k*-th user, with the PCE and CEEs of 5%, 10%, and 20% with 4-QAM and 16-QAM strategies. The AWGNk is $\omega_{k}$. The UWA image transmission BER is $10^{-4}$. When PSR values are 90%, 80%, 70%, 60%, 50%, and 40%, the AWGNk values are 0.00630, 0.012, 0.0179, 0.0235, 0.0285, and 0.0353, and 0.00089, 0.00176, 0.00263, 0.00343, 0.0042, and 0.0054 when using 4-QAM and 16-QAM with CEE of 10%, respectively. With the 4-QAM strategy, the PSR of 90% outperformed the PSR of 40% as the AWGNk decreased by 0.0290. The 4-QAM outperformed 16-QAM, under a PSR of 60%, with the AWGNk decreasing by 0.0201. At PSR of 60%, the PCE outperformed the CEEs of 20% under 4-QAM and 16-QAM with the AWGNk decreasing by 0.0111 and 0.0051, respectively. As the CEEs increased, the AWGNk values decreased.

The simulation results show that the proposed GFDM-based low-power UWA image transceiver is a suitable image signal transmission method for future advanced underwater image sensor network technology.

## 4. Conclusions

This paper proposes a transceiver scheme for UWA image communication that integrates advanced transmission technologies such as GFDM-based modulation, LDPC code, adaptive 4-QAM and 16-QAM, and a power assignment mechanism to transmit image sensor signals over UW channels. Simulation results show that the GFDM-based UWA image transceiver with the PCE can have the same transmission BER with lower SNR than the transceiver with CEE of 20%. For a BER of $2.73\times 10^{-5}$, 4-QAM outperformed 16-QAM, with an SNR increase of 12.04 dB. Compared with 16-QAM, 4-QAM showed better PSNR performance of the received UW image signals using GFDM-based UWA image transceiver with the PCE, the CEEs of 5%, 10%, and 20%, and with SNR gains of approximately 7.08 dB, 7.22 dB, 7.99 dB, and 12.13 dB, respectively, when BER was $10^{-4}$. As the CEE increased, the SNR gains with 4-QAM were better than those with 16-QAM.

When using 4-QAM and 16-QAM strategies, with CEE of 10%, the PSR of 90% outperformed the PSR of 40%, with an AWGNk increase of 0.0290 and 0.00451, respectively. The 4-QAM outperformed 16-QAM, under a PSR of 60%, with the AWGNk decreasing by 0.0201. The PCE outperformed the CEEs of 20%, under 4-QAM and a PSR of 60%, with the AWGNk decreasing by 0.0111. The PCE outperformed the CEEs of 20%, under 16-QAM and a PSR of 60%, with the AWGNk decreasing by 0.0051. As the CEE increased, the AWGNk fading decreased.

The PSNR of the received UW image signal 1, 2, and 3, using 4-QAM, with a CEE of 10%, transmission BERs of $10^{-4}$, were 62.39 dB, 44.46 dB, and 45.64 dB, respectively. The PSNR of the received UW image signal 1, 2, and 3, using 16-QAM with a CEE of 10%, transmission BERs of $10^{-4}$, were 71.63 dB, 48.79 dB, and 49.87 dB, respectively. The received UW images have high PSNR values and high image resolutions. The proposed GFDM-based UWA transceiver could meet the transmission BER quality-of-service requirements for UW image sensor applications by maximizing the transmission throughput or minimizing power consumption.

## Figures and Tables

**Table 1:** Simulation parameters of the proposed GFDM-based low-power UWA image transceiver.

Technology	Technology Characteristics
GFDM modulation	Michailow et al. [15]
Channel model	Chitre et al. [31]
Number of subcarriers (B)	128
Number of subsymbols (C)	9
Filter method	Root-raised cosine
Roll-off factor	0.1
Channel bandwidth	20 kHz
Adaptive modulation	4-QAM and 16-QAM
Channel coding	(2000, 1000) LDPC code encoder with a code rate of 1/2, a column weight of 3, a row weight of 6
UW transmission media	Image signal
Power weighting factors	1/10, 2/10,…,10/10
BER limits for image transmission	10^−4^

## Data Availability

Not applicable.
